# Identification of small proline‐rich protein 1B (SPRR1B) as a prognostically predictive biomarker for lung adenocarcinoma by integrative bioinformatic analysis

**DOI:** 10.1111/1759-7714.13836

**Published:** 2021-01-26

**Authors:** Zihe Zhang, Ruifeng Shi, Songlin Xu, Yongwen Li, Hongbing Zhang, Minghui Liu, Guangsheng Zhu, Chen Chen, Zhenhua Pan, Hongyu Liu, Jun Chen

**Affiliations:** ^1^ Department of Lung Cancer Surgery Tianjin Medical University General Hospital Tianjin China; ^2^ Tianjin Key Laboratory of Lung Cancer Metastasis and Tumor Microenvironment Tianjin Lung Cancer Institute, Tianjin Medical University General Hospital Tianjin China; ^3^ Quantitative Biomedical Research Center, Department of Population and Data Sciences University of Texas Southwestern Medical Center Dallas Texas USA; ^4^ Department of Thoracic Surgery First Affiliated Hospital, School of Medicine, Shihezi University Shihezi China

**Keywords:** non‐small cell lung cancer (NSCLC), small proline‐rich protein 1B (SPRR1B), biomarker predictor, prognosis

## Abstract

**Background:**

With the ongoing development of targeted therapy and immunotherapy in recent years, the overall five‐year survival rate of NSCLC patients has not improved, and the search for novel diagnostic and prognostic markers for lung adenocarcinoma continues.

**Methods:**

Lung adenocarcinoma (LUAD) gene expression data and relevant clinical information were obtained from the TCGA. Hub genes were identified with weighted gene co‐expression network analysis (WGCNA) and protein–protein interaction network (PPI). Survival analyses were also performed using GEPIA. The 536 LUAD patients were divided into two groups according to the SPRR1B expression level and analyzed by gene set enrichment analysis (GSEA) and verified by immunoblotting. The effects of SPRR1B on cell proliferation and cell metastasis and apoptosis were evaluated by 5‐ethynyl‐2′‐deoxyuridine (EdU) staining, colony formation assay, transwell migration and invasion assay, and flow cytometry, respectively.

**Results:**

A total of 2269 DEGs were analyzed by WGCNA and five hub genes (CCK, FETUB, PCSK9, SPRR1B, and SPRR2D) were identified. Among them, SPRR1B was selected as one of the most significant prognostic genes in LUAD. SPRR1B was found to be highly expressed in lung adenocarcinoma cells compared with that in normal bronchial epithelial cells. In addition, silencing of SPRR1B could inhibit the cell proliferation, invasion, and migration of lung adenocarcinoma cells, but induced cell apoptosis and G2/M phase arrest in vitro. The result of GSEA and immunoblotting revealed that SPRR1B activated the MAPK signaling pathway involved in the proliferation and metastasis of lung cancer.

**Conclusions:**

Our findings demonstrate that SPRR1B may function as a prognosis predictor in lung adenocarcinoma.

## Graphical abstract

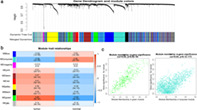

## INTRODUCTION

As one of the most common malignant tumors, non‐small cell lung cancer (NSCLC) is characterized by high morbidity and mortality rates.^1^ The five‐year survival rate of lung cancer patients varies from 4% to 17% depending on the stage and regional differences.^2^ Traditionally, lung cancer is divided into two main histological types: small cell lung cancer (SCLC) (15% to 25% of lung cancers) and NSCLC (75% to 85% of lung cancers). In addition to the traditional methods, prognostic methods such as clinicopathological staging, tumor biology and molecular genetics, perioperative factors and postoperative adjuvant therapy are needed to identify new biological or pathological indicators related to the prognosis of lung cancer. With the development of genome technology, bioinformatics has become increasingly popular for gene expression profile analysis to study the molecular mechanisms of disease and to discover disease‐specific biomarkers.^3^

The SPRR1B gene located in 1q21.3, also known as SPRR1, belongs to the SPRR family. SPRR1B consists of 89 amino acids, and its molecular weight is approximately 9888 Da.^4^ SPRR1B is the envelope protein of keratinocytes which forms an insoluble layer under the plasma membrane by transglutaminase cross‐linking with the membrane protein. This protein is rich in proline and contains multiple tandem amino acid repeats which can also be detected in the skin, conjunctiva, and oral cavity.^5^

Weighted gene co‐expression network analysis of (WGCNA) is a crucial method for understanding gene function and association with the genome‐wide expression profile.^6^ The co‐expression module is constructed and is used to study the relationship between basic modules and clinical features. In addition, a functional enrichment analysis of these co‐expressed genes based on relevant modules has been carried out.^7^ This approach provides great insight for predicting the function of co‐expressed genes and discovering genes that play a key role in human diseases. The results of WGCNA and differential gene expression analysis were combined to improve the recognition ability of highly related genes as candidate biomarkers.

In this study, differential gene expression analysis and WGCNA analysis were performed on the mRNA expression data of LUAD in the TCGA database. Functional enrichment and protein‐protein interaction (PPIs) analysis were combined with the Lasso‐Cox regression model analysis to identify and analyze the genes related to the prognosis of lung cancer. SPRR1B was determined to be a prognostic marker of lung adenocarcinoma. We also found that it plays a role in cell proliferation, invasion, migration, the cell cycle, and apoptosis. This study provides a potential basis for understanding the etiology and potential molecular events of lung cancer.

## METHODS

### mRNA expression dataset and download

We obtained The Cancer Genome Atlas Lung Adenocarcinoma (TCGA‐LUAD) gene expression data from TCGA (https://portal.gdc.cancer.gov/), which contains data related to 585 samples. The dataset was obtained using Illumina HiSeq2000, and the format of the data was FPKM. All mRNA expression data on LUAD and corresponding clinical information were downloaded by the R package TCGAbiolinks.

### Identification and enrichment of the hub module used by weighted gene co‐expression network analysis

A co‐expression network was constructed by using R package WGCNA,^6^ and the average linkage and Pearson's correlation values were calculated. To build a scale‐free network, we chose β = 3 (scale‐free R^2^ = 0.75) as the soft‐thresholding power. The dynamic hybrid cutting method was used to construct the hierarchical clustering tree. Each leaf on the tree represents a gene, and genes with similar expression data are closely linked to form a branch of the tree and represent a gene module. Pearson's test was used to clarify the correlation between the module eigengenes of tumor and normal tissue; *p* < 0.05 indicates significance. Modules with high correlation coefficients that were identified as candidates for the relationship between cancer and normal tissues were selected for subsequent analysis, which was calculated following the study by Wang et al.^8^

### Construction of PPI and screening of hub genes

The network of PPIs was constructed using STRING (https://string-db.org/),^9^ an online search tool for retrieving interacting genes. By using the Monte Carlo Collisions (MCC) algorithm of the CytoHubba plugin,^10^ genes having the top 100 MCC values were considered to be hub genes. Cytoscape (https://cytoscape.org/)^11^ was used to visualize results.

### Univariate Cox analysis and Lasso‐Cox regression analysis

Univariate regression analysis was used to evaluate the association between genes and the overall survival (OS). The Lasso‐Cox regression model was used for variable selection to calculate the most useful prognostic genes.^12^ The dotted line on the left indicates the best model. The Hub genes screened by PPIs and Lasso‐Cox regression analysis were intersected using a Venn online tool (http://bioinformatics. psb.ugent.be/webtools/Venn/).

### Kaplan–Meier analysis and Oncomine meta‐analysis

Microarray datasets on tumors and normal tissues were obtained from the Oncomine Cancer Microarray database (Oncomine, https://www.oncomine.org/resource/login.html) to perform meta‐analysis.^13^ Kaplan–Meier analyses were obtained from Gene Expression Profiling Interactive Analysis (GEPIA, http://gepia.cancer-pku.cn/).

### Differentially expressed gene analysis and gene set enrichment analysis (GSEA)

TCGA‐LUAD gene expression data were downloaded by R package TCGAbiolinks, and the format of the data was “counts”. The R package DESeq2 were applied to find the differentially expressed genes (DEGs), which were visualized as a volcano plot by R package ggplot2. GSEA is a calculation method that determines whether a set of basically defined genes shows statistically significant differences between two biological states.^14^ The enrichment pathway was analyzed by R package clusterProfiler and visualized using the R package ggplot2; the color and size of the dots represent the *p*‐value and numbers of genes in the pathway, respectively.

### Cells and cell culture

The A549, H1975, H1650, and BEAS‐2B cell lines were obtained from American Type Culture Collection (Manassas). The H1299 and PC9 cell lines were obtained from the Cell Bank of the Chinese Academy of Sciences. All cell lines were maintained at 37°C in RPMI 1640 medium supplemented with 10% fetal bovine serum (FBS; Gibco).

### RNA extraction, reverse transcription and quantitative real‐time PCR

After washing with phosphate‐buffered saline (PBS), TRIzol (Thermo Fisher Scientific) and the RNeasy Mini Kit (Qiagen) were used to extract the total RNA. Nanodrop2000 was used to detect the RNA concentration. Then, the M‐MLV reverse transcriptase kit (Promega) was used to synthesize cDNA according to the protocols. ABI SYBR Green Master Mix (Thermo Fisher Scientific) was used for real‐time quantitative PCR analysis through the ABI7500 real‐time PCR system. The transcript level was normalized to the housekeeping gene glyceraldehyde‐3‐phosphate dehydrogenase (GAPDH) and analyzed by using the relative quantitative 2^‐ΔΔCt^ method. All gene primers were purchased from BGI. The primers used are listed as followed: SPRR1B: forward: 5′‐TCCCCTATCCCATTCTGCGT‐3′, reverse: 5′‐GCAGCTGAAACTAGCTCTGG; GAPDH: forward: 5′‐GGAGCGAGATCCCT CCAAAAT‐3′, reverse: 5′‐GGCTGTTGTCATACTTCTCATGG‐3′.

### RNA interference

SPRR1B knockdown was performed by transfecting cells with independent siRNA candidate Si‐SPRR1B‐2. Cells were transfected using Lipofectamine 3000 (Life Technologies) according to the manufacturer's protocol. Negative siRNA Si‐NC was used as the mock control. SPRR1B expression was then detected by real‐time PCR and immunoblotting after 48 h transfection. All siRNAs were purchased from RiboBio. The sense sequences of siSPRR1B were as follows: si‐SPRR1B‐2 (GCAGCAGGTGAAACAGCCT).

### 5‐ethynyl‐2′‐deoxyuridine (EdU) staining

A Cell‐Light EdU stain kit was purchased from RiboBio. The cells were incubated with EdU at 37°C for 2 h, then washed twice with PBS, fixed with 4% paraformaldehyde for 30 min, permeated with 0.5% Triton X‐100 for 15 min, and washed with 0.5% Triton X‐100. Then, the proliferated cells were stained with EdU and Hoechst 33342 nucleic acid stain, washed again in PBS, and analyzed with a fluorescence microscope.

### Colony formation assay

After SiRNA transfection, cells were seeded into six‐well plates, 500 cells per plate, and incubated for 14 days. Plates were washed twice with cold PBS and stained with 0.5% crystal violet, stained for 30 min at room temperature, and then washed with cold PBS. The number of colonies was counted.

### Cell cycle and apoptosis assay

After 48 h of transfection with siRNA, the cell pellet was collected and washed twice with cold PBS. The cells were fixed with cold 70% ethanol at room temperature overnight. The supernatant was removed by centrifugation the next day and washed with PBS again. The cells were then stained with propidium iodide (PI; BD Biosciences). To detect apoptosis, the cell pellet was collected, washed twice with cold PBS, combined with Annexin V‐FITC and PI, and was stained for 15 min at room temperature. Finally, an FACS Calibur flow cytometer (Beckman Coulter) was used to analyze the cell cycle and apoptosis. The cell cycle results were analyzed using Kaluza v2.1.1 software.

### Transwell migration and invasion assay

For the cell invasion assay, 100‐μl 30% Matrigel was coated on the upper surface of the polycarbonate membrane with a transwell filter (Corning). After 48 h of SiRNA transfection, cells in 2 × 10^5^ serum‐free RPMI1640 were seeded in the upper chamber, and 600‐μl of RPMI1640 containing 10% FBS was added to the lower chamber. The cells were cultured at 37°C in a humidified incubator with 5% CO_2_. After 24 h of incubation, the cells that migrated to the underside of the filter were fixed with methanol for 15 min, washed with PBS, stained with 0.1% crystal violet for 30 min, and washed again with PBS. The process for the cell migration assay was similar to that of the cell invasion assay, except that the upper chamber was not coated with Matrigel, and the number of cells seeded in the upper chamber was 1 × 10^5^. A Nikon TE2000 microscope (Nikon) was used to image and count five randomly selected fields of view. The average value in three independent experiments was obtained.

### Immunoblotting

Immunoblotting was performed as previously described^15^ using Rabbit anti‐SPRR1B (1 μg/μl,Abcam), Rabbit anti‐GAPDH, Rabbit anti‐MAPK, and Rabbit anti‐p‐MAPK (1:1000, Cell Signaling Technology).

### Statistical analysis

All data were analyzed by using GraphPad Prism 8.0.1 and IBM SPSS Statistics 26 (SPSS). Student's *t*‐test and Chi‐square test were used to determine the significance of the difference between the two groups. The overall survival curve was drawn according to the Kaplan–Meier method, and the log‐rank test was used to compare the survival distributions in non‐crossover trend. At the level of *p* < 0.05, all differences were considered to be statistically significant.

## RESULTS

### Identification of key co‐expression modules and genes in lung adenocarcinoma based on WGCNA

**FIGURE 1:**
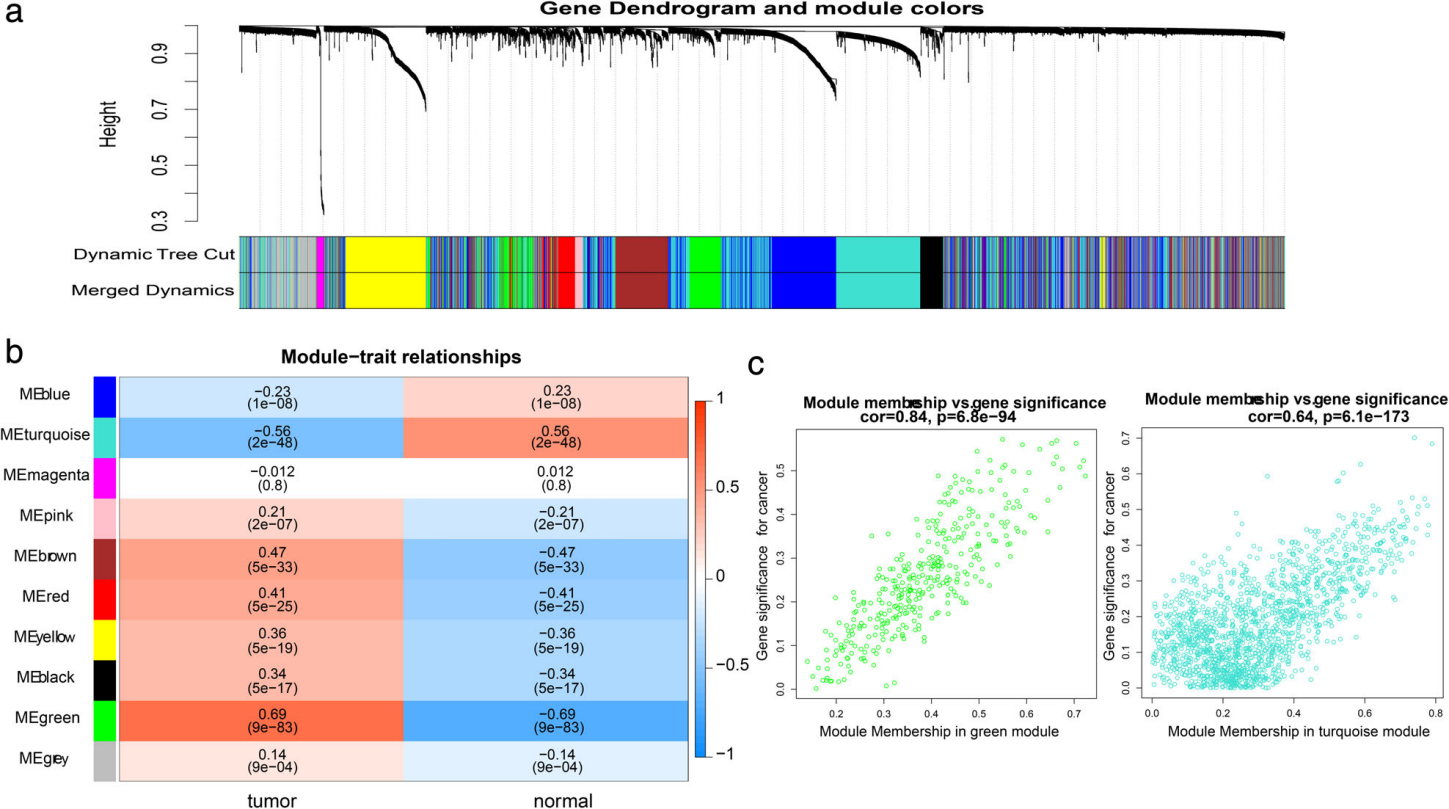
Identification of modules representing the associations between lung adenocarcinoma and normal tissue. (a) Hierarchical clustering and dynamic cutting were used to construct a hierarchical cluster tree involving nine modules of co‐expression. (b) Heatmap showing the module correlations of between lung adenocarcinoma and normal tissue. (c) The scatter plot represents all genes in green and turquoise modules, and one dot represents a gene

In this study, data on 536 lung adenocarcinoma tissue samples and 49 normal tissue samples from TCGA were included. We constructed a gene co‐expression network of 5000 genes using the WGCNA R package, and selected 3 as the soft threshold power parameter (scale‐free R^2^ = 0.9) to ensure a scale‐free network. Then, hierarchical clustering and dynamic cutting were used to construct a hierarchical cluster tree involving nine modules of the co‐expression (gray modules were excluded because they were not assigned into any cluster), in which each tree branch represented a module; each leaf in the branch represented one gene; and genes with similar expression patterns and similar functions formed a color module (Figure 1(a)). We then analyzed the interaction between the nine modules and plotted the network heat map and assessed the association between cancerous and normal tissue in each module (Figure 1(b)). Among the nine modules, green and turquoise modules were highly correlated between normal and cancerous tissues (Figure 1(c)). The 2269 DEGs were selected from these two modules for further analysis.

### Screening the most useful prognostic genes by combining PPIs network construction and the Lasso‐Cox regression model

To explore the potential relationships among the 2269 genes, we tried to construct a network of PPIs by using STRING, which is an online search tool for retrieving interacting genes. In this network, 680 nodes with 2673 joint‐edges have been integrated.

**FIGURE 2:**
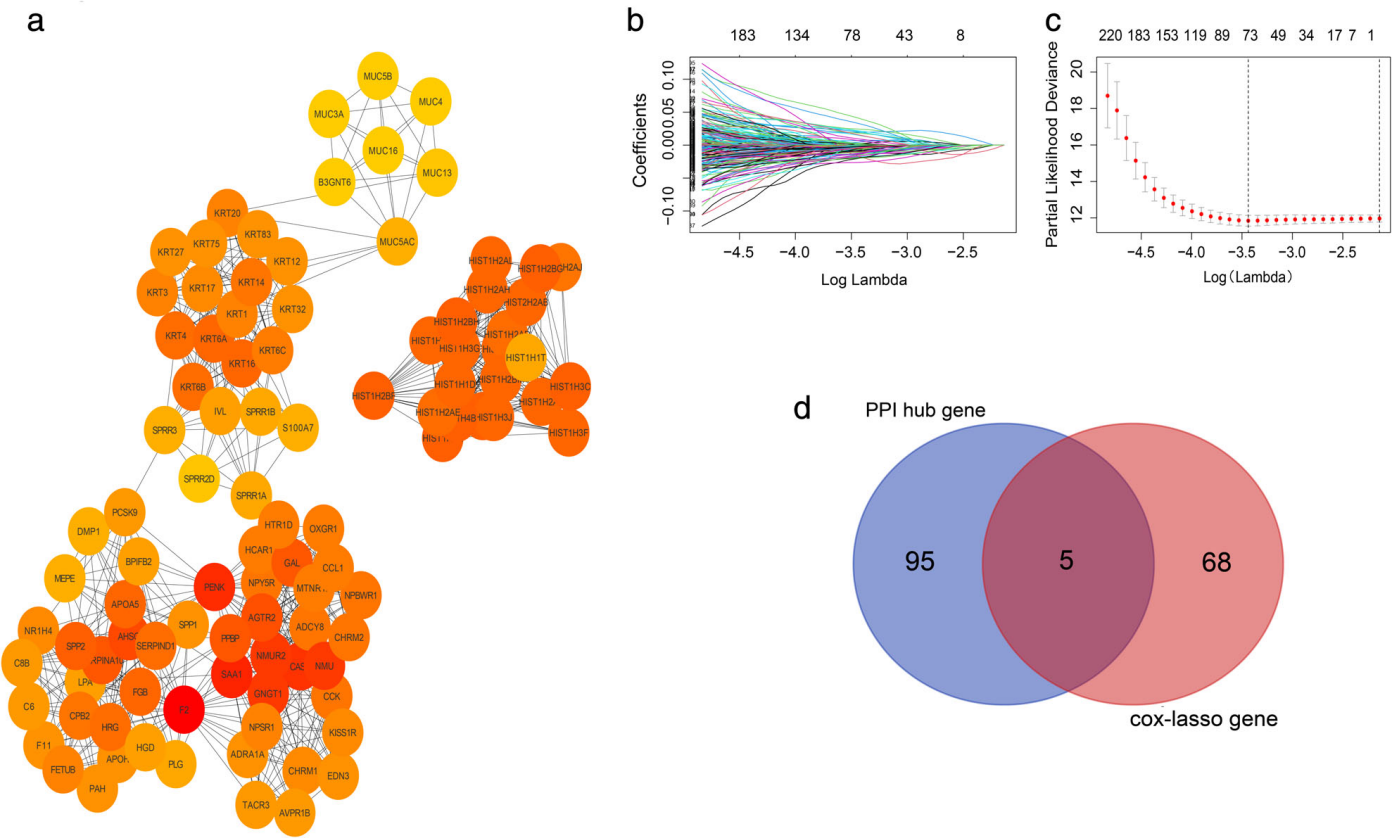
Screening the hub genes related to prognosis from green and turquoise modules. (a) The 100 genes of green and turquoise modules with the highest scores in the PPI network obtained by using the MCC algorithm of the CytoHubba plugin. (b) The Lasso coefficient of the most useful prognostic gene through Lasso‐Cox regression analysis. (c) Cross‐validation in the Lasso model. The dotted line on the left is the best model, and the simplest model is on the right. (d) The Venn diagram represents the hub gene that was selected based on the overlap of the PPI network and the Lasso‐Cox regression model

The hub genes were selected from the PPI network by using the MCC algorithm of the CytoHubba plugin, and we then screened out the top 100 hub genes with the highest scores on the MCC (Figure 2(a)). At the same time, by performing a Univariable Cox regression analysis of the 2269 genes in green and turquoise modules and on the OS of these tissue samples, we found that 438 genes had a significant statistical correlation with the OS. Next, these 438 genes were used to construct the Lasso‐Cox regression model to calculate the genes with the best prognosis, from which 73 genes were filtered out (Figure 2(b) and (c)). Hence, by combining these two analysis methods, five genes were selected as the central genes considered to be prognosis‐related hub genes (Figure 2(d)), including CCK, FETUB, PCSK9, SPRR1B, and SPRR2D.

### SPRR1B is highly expressed in lung adenocarcinoma and serves as a prognostic biomarker of lung adenocarcinoma

**FIGURE 3:**
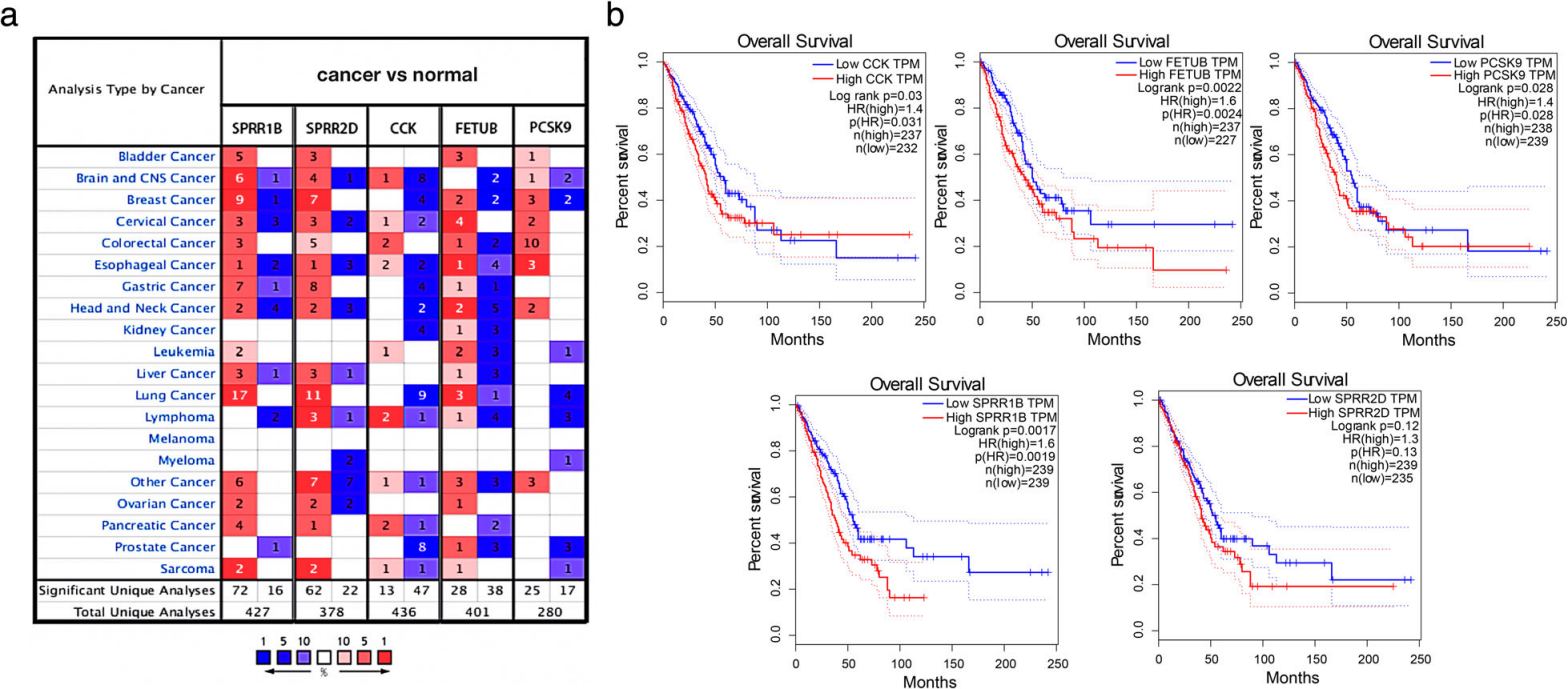
Oncomine meta‐analysis and Kaplan–Meier analysis selected the best prognostic gene. (a) Analysis of the expression differences of five hub genes in most major types of cancers and respective normal tissues through the Oncomine meta‐analysis. Red represents high expression, and blue represents low expression. (b) Kaplan–Meier analysis among five hub genes. Log rank *p* < 0.05 was considered to be statistically significant

To further investigate the roles of these five hub genes in cancer, especially lung cancer, we used Oncomine to conduct a meta‐analysis of the datasets of these genes in most major types of cancers and in their adjacent normal tissues (Figure 3(a)). Accordingly, we found that the expressions of SPRR1B and SPRR2D in multiple tumor data sets were higher in tumor tissues than in normal tissues (72 vs. 16 and 62 vs. 22, respectively), and the data sets with high expression in lung cancer were significantly more than those of other types of cancer. Next, GEPIA was used to perform a Kaplan–Meier analysis of these five hub genes (Figure 3(b)). FETUB and SPRR1B showed statistical significance in this analysis, which indicates that the high expression of these genes has a close correlation with the prognosis of patients. The above results demonstrated that SPRR1B is highly expressed in lung cancer and is closely related to poor prognosis. Thus, we focused on SPRR1B as a prognostic biomarker candidate for further validation.

### SPRR1B is highly expressed in lung adenocarcinoma cells

**FIGURE 4:**
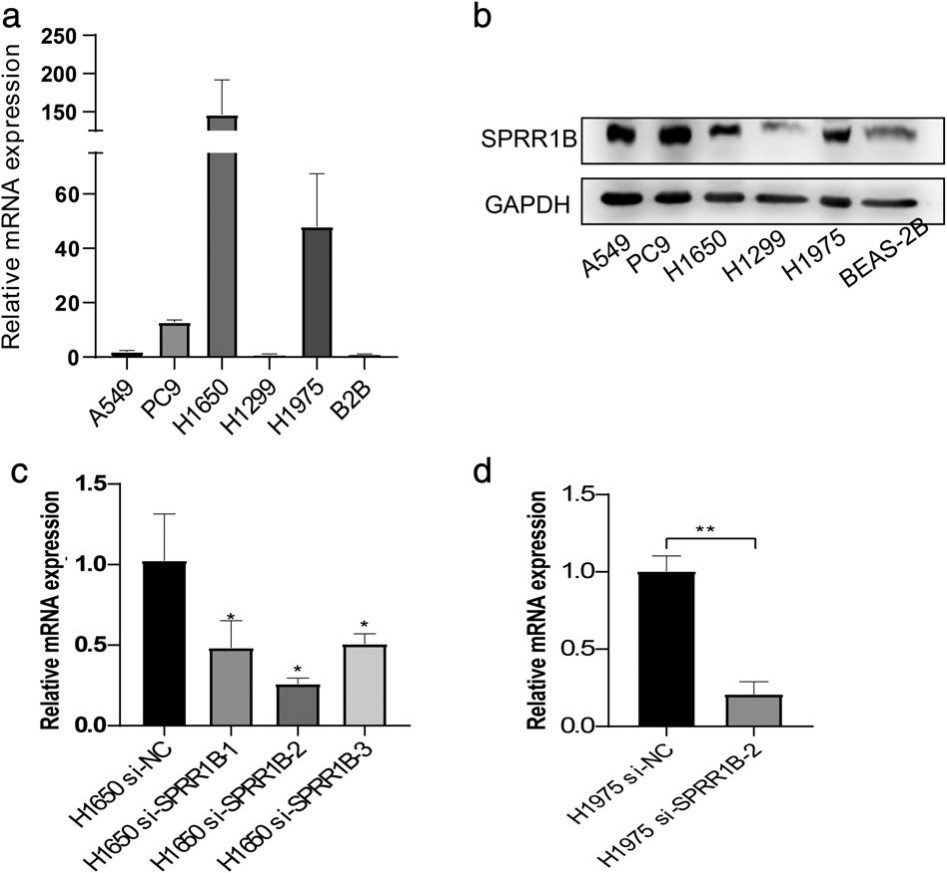
Differences in SPRR1B expression between lung adenocarcinoma cell lines and normal lung epithelial cell. (a, b) The mRNA and protein levels of SPRR1B in different lung adenocarcinoma cells compared with those of the normal lung epithelial cells. (c, d) H1650 and H1975 cells were transfected with si‐SPRR1B and control‐siRNA. mRNA levels were analyzed using qRT‐PCR. Data is presented as mean ± SD (*n* = 3 independent experiments). * indicates *p* < 0.05, ** indicates *p* < 0.001

To clarify the changes in the expression of SPRR1B in lung adenocarcinoma cell lines, we used five different lung adenocarcinoma cell lines (A549, PC9, H1975, H1650, H1299) and a normal human bronchial epithelial cell line (BEAS‐2B). As shown in Figure 4(a), the mRNA level of SPRR1B in A549, PC9, H1975, and H1650 showed higher expression levels (1.954‐fold *p* < 0.05, 12.85‐fold *p* < 0.0001, 146.0‐fold *p* < 0.01, and 47.9‐fold *p* < 0.05) compared with that of BEAS‐2B. Simultaneously, the Western blotting result showed a similar trend (Figure 4(b)). To further investigate the potential function of SPRR1B in lung adenocarcinoma cells, siRNAs were used to interfere with SPRR1B expression in H1650 and H1975 cells. Si‐SPRR1B‐2 was finally chosen because it has the highest interference efficiency (Figure 4(c) and (d)).

### Silencing of SPRR1B inhibited cell proliferation of lung adenocarcinoma cells

**FIGURE 5:**
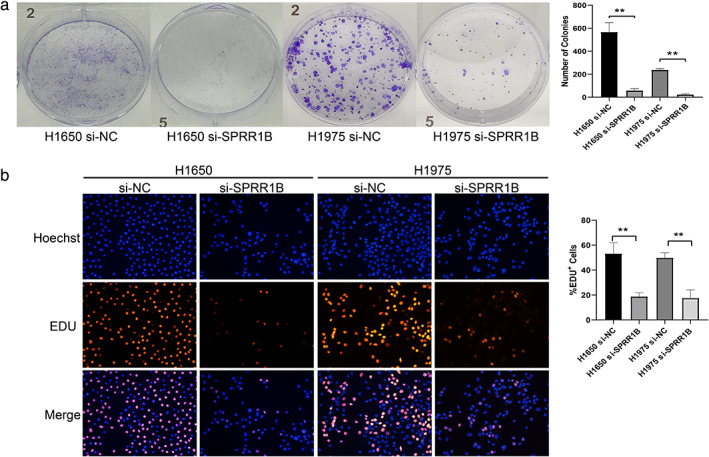
SPRR1B downregulation enhanced the inhibition of cell proliferation. (a) Colony‐formation assays were performed to show the influence of Si‐NC and Si‐SPRR1B on the proliferation between H1650 and H1975 cells. (b) 5‐ethynyl‐2′‐deoxyuridine (Edu) staining assays were used to detect the effects of each group on proliferation. Data are presented as mean ± SD (*n* = 3 independent experiments) **p < 0.01, compared with the control group

To investigate the effect of SPRR1B on the proliferative capability of lung adenocarcinoma cells, the cell proliferative capability was examined using the colony formation assay and EdU assay after transfection with SPRR1B siRNA for 48 h. The result (Figure 5(a)) showed that the proliferation of both cell lines was obviously inhibited after SPRR1B knockdown in H1650 and H1975 cells (*p* < 0.001 and *p* < 0.0001, respectively). EdU proliferation assay also revealed that silencing SPRR1B decreased the number of EdU‐positive cells (*p* < 0.0001 and *p* < 0.0001, respectively (Figure 5(b)). These data demonstrated that silencing SPRR1B inhibited cell proliferation of lung adenocarcinoma cells.

### SPRR1B **knockdown induced** G2 **/M phase arrest and cell apoptosis in lung adenocarcinoma cells**

**FIGURE 6:**
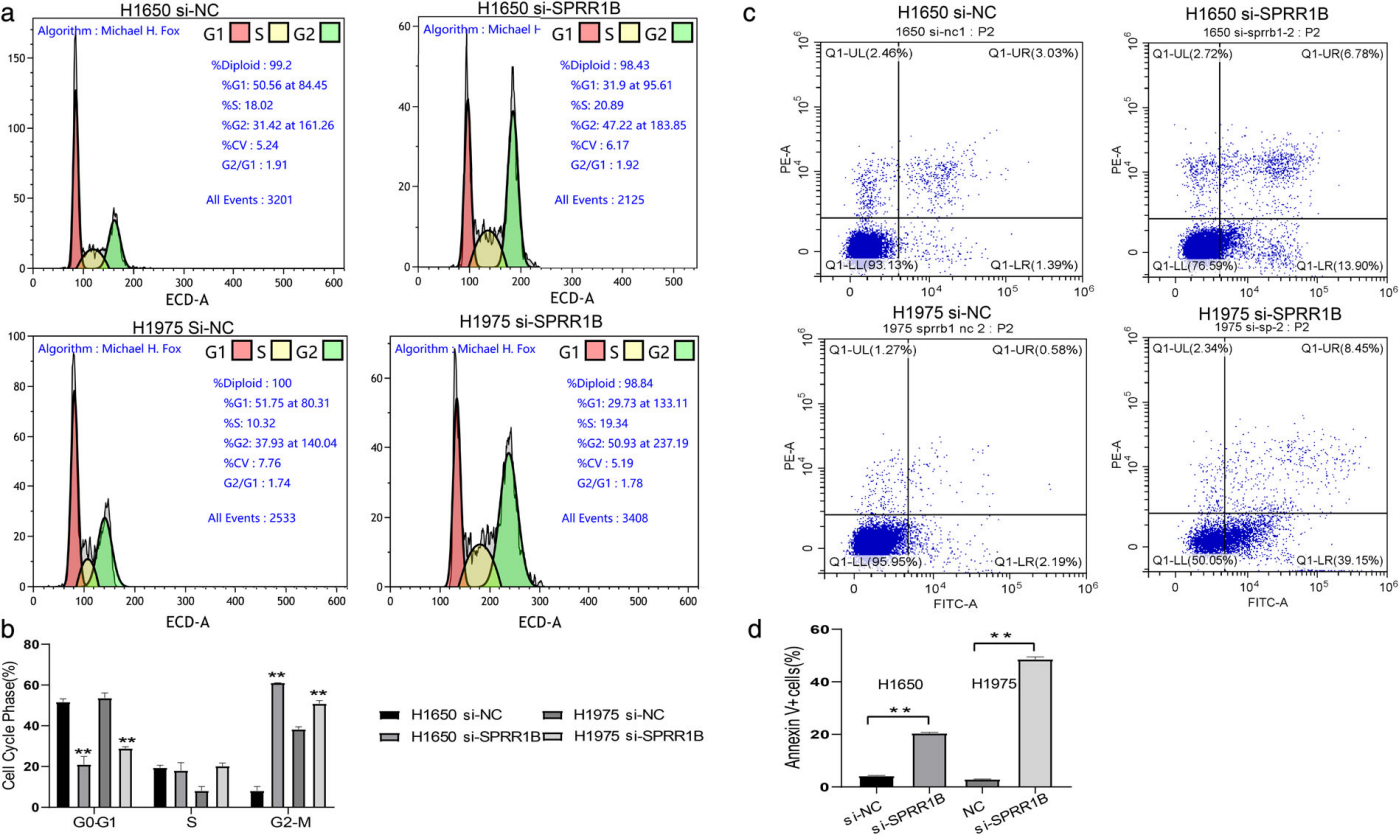
SPRR1B inhibition induced G2/M‐phase arrest in lung adenocarcinoma cell lines and enhanced apoptosis. H1650 and H1975 were transfected with si‐NC and si‐SPRR1B. The cell cycle (a, b) and apoptosis (c, d) were assessed after 48 h. Data are presented as mean ± SD (*n* = 3 independent experiments),**p < 0.001

Next, cell cycle distribution was assessed by PI staining and flow cytometry analysis. As illustrated in Figure 6(a), SPRR1B inhibition significantly decreased the percentage of cells in the G0/G1 phase (51.67% to 21.02%, *p* < 0.01 and 53.63% to 28.91%, *p* < 0.01) and increased the percentage of cells in the G2 phase in both H1650 and H1975 lung adenocarcinoma cell lines (8.078% to 61.09%, *p* < 0.01 and 38. 03% to 50.87%, *p* < 0.01) In addition, Annexin V/PI staining was used to assess cell apoptosis. After SPRR1B knockdown for 48 h, changes in the cell apoptosis rate were assessed by flow cytometry (Figure 6(b)). Compared with the control groups, the apoptosis rates increased 16.25% and 45.70% (*p* < 0.01 and *p* < 0.01, respectively). These results demonstrate that SPRR1B inhibition can enhance cell apoptosis in lung adenocarcinoma cells.

### SPRR1B knockdown inhibited cell migration and invasion of lung cancer cells in vitro

**FIGURE 7:**
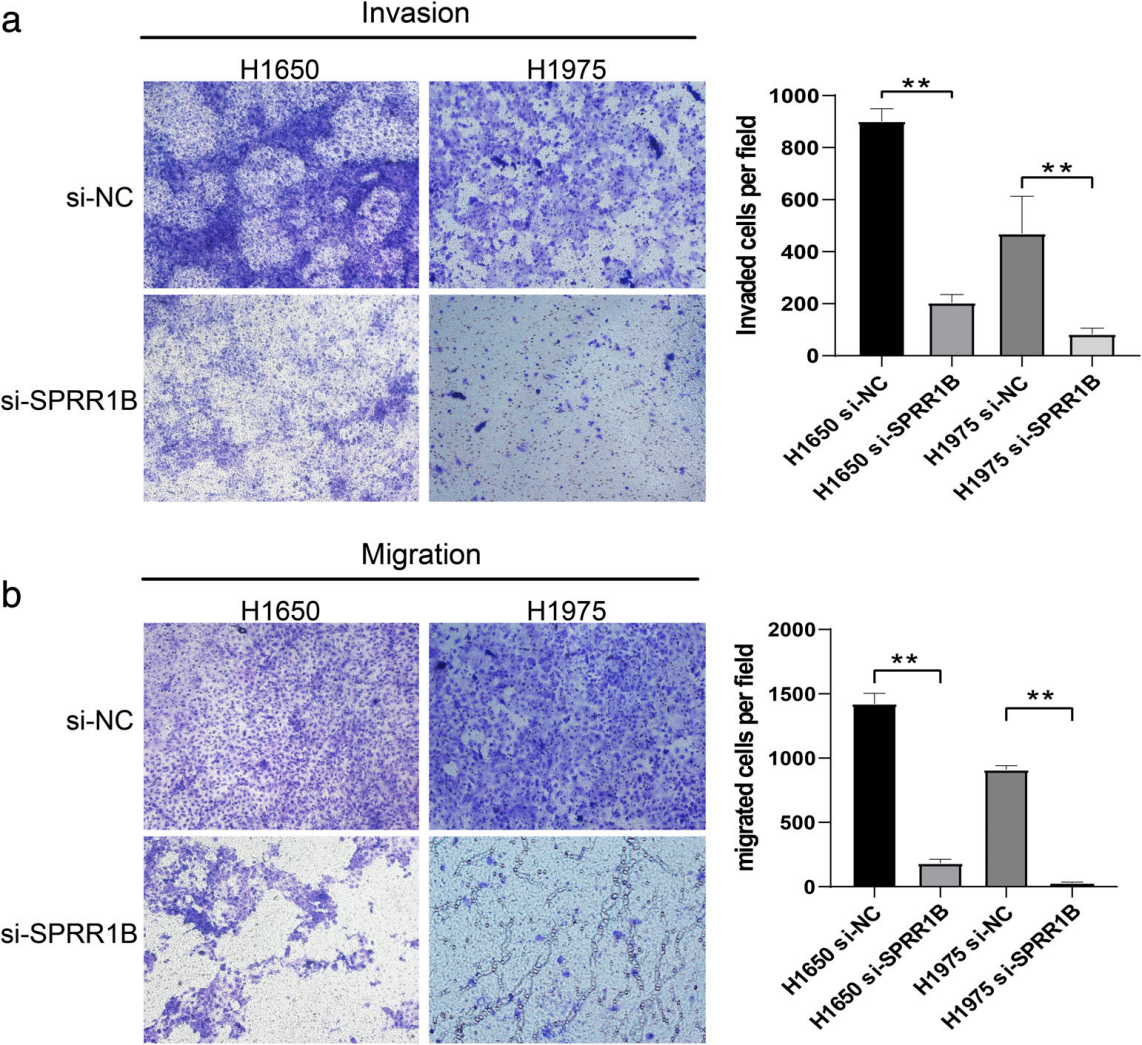
SPRR1B knockdown inhibited lung cancer cell migration and invasion in vitro. H1650 and H1975 were transfected with si‐NC and si‐SPRR1B for 48 h. Invasion and migration functions of different groups were detected by using transwell assay. Data are presented as mean ± SD (*n* = 3 independent experiments) **p < 0.001

To more carefully elucidate the role of SPRR1B in cell metastasis of lung adenocarcinoma cells, cell migration and invasion were confirmed in vitro through transwell assay. Results revealed that SPRR1B knockdown significantly inhibited cell migration (901.0 to 204.0, *p* < 0.01 and 469.7 to 83.0, *p* = 0.01, Figure 7(a)) and invasiveness (1423.0 to 182.3, *p* < 0.01 and 909.3 to 29.7, *p* < 0.01) in H1650 and H1975. Compared with the control, these results indicated that SPRR1B suppresses the migration and invasion capacity of H1650 and H1975 cells in vitro.

### SPRR1B affects the cell phenotype by regulating the MAP kinase signaling pathway

**FIGURE 8:**
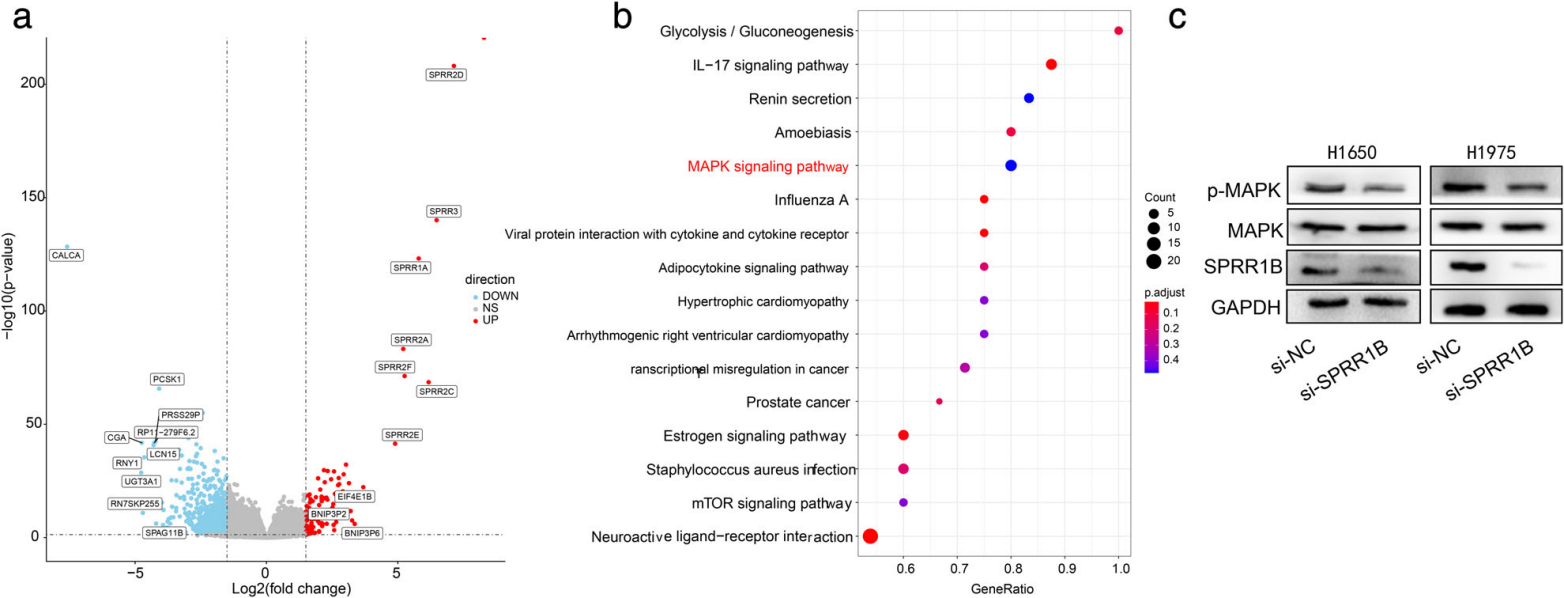
DEG screening and GSEA enrichment. (a) Volcano plot of mRNA expressions from high to low expression in SPRR1B groups. The x‐axis represents the log2 fold‐change, and the y‐axis represents–log10 *(p‐*value). The horizontal and vertical dotted lines represent the filter criteria. Red dots represent upregulated genes, and blue dots represent downregulated genes. (b) Gene set enrichment analysis (GSEA) was used to analyze the signaling pathway enrichment among DEGs. (c) H1650 and H1975 were transfected with si‐NC and si‐SPRR1B for 48 h to detect whether the MAP kinase signaling pathway is activated by immunoblotting. Three independent experiments were performed

To gain further insights into the mechanism of SPRR1B in lung adenocarcinoma, 536 LUAD patients were divided into high and low expression groups according to the median expression levels of SPRR1B. When DEGs in the two groups were analyzed by using DESeq2, the number of significant DEGs between the two groups was 2265, where 262 genes and 2003 genes were up‐ and downregulated, respectively (Figure 8(a)). Then, DEGs were enrichened using gene set enrichment analysis (GSEA). The result indicated that the mitogen‐activated protein (MAP) kinase signaling pathway was highly enriched (Figure 8(b)). The MAP kinase signaling pathway can regulate cell growth, differentiation, apoptosis, cell death, and other physiological processes.^16^ Therefore, we used the H1650 and H1975 cell lines to explore whether SPRR1B modulates the cell phenotype by regulating the MAP kinase signaling pathway. The western blot results showed that the expression level of phosphorylated MAP kinase in SPRR1B knockdown cells were significantly downregulated (Figure 8(c)), which implies that SPRR1B may affect the phenotype of lung adenocarcinoma cells by regulating the MAP kinase signaling pathway.

## DISCUSSION

With the development of molecular targeted therapy and immunotherapy drugs, the OS rates of lung cancer have improved significantly in recent years.^17^, ^18^ However, the available drug therapies are not suitable for all lung cancer patients. Meanwhile, as the use of drugs increases over time, drug resistance has been reported,^19^ which means that the patient's prognosis is still limited. The identification of novel and effective prognostic biomarkers of NSCLC is of vital importance. In the present study, the module associated with lung adenocarcinoma and adjacent normal tissues was identified by applying WGCNA. Then, five hub genes associated with prognosis were selected and combined with PPIs and Lasso‐Cox regression analysis. SPRR1B was determined to be a prognostic marker of lung adenocarcinoma, based on Oncomine meta‐analysis and Kaplan–Meier analysis, as its expression increased in lung adenocarcinoma tissues and cell lines. SPRR1B knockdown can inhibit lung adenocarcinoma cell proliferation, migration, and invasion, and it induced the G2/M phase arrest in vitro.

As a member of the SPRR family, SPRR1B has been studied in various diseases, including cancers, conjunctiva disease,^20^and cutaneous disease.^21^ Previous studies have suggested that SPRR1B is overexpressed in oral squamous cell carcinoma stem‐cell‐like cells, thereby affecting cell growth.^22^ SPRR1B has also been identified as one of the predictive indicators for the prognosis of patients with pancreatic cancer^23^ or metastatic transformation of melanoma.^24^ In head and neck squamous cell carcinoma (HNSCC), SPRR1B affects HNSCC angiogenesis by targeting MDA‐9/Syntenin, thereby affecting tumor invasion and differentiation.^25^ SPRR1B is an early biomarker for squamous differentiation of the airway epithelium. Downregulation of SPRR1B expression occurs with malignant transformation.^26^ Carcinogens, smoking, and phenylmercapturic acid (PMA) stimulate the transcription of SPRR1B, thereby promoting airway squamous metaplasia.^27^ Moreover, the available studies showed that SPRR1B was overexpressed in lung squamous cell carcinoma tissue.^28^ Research on SPRR1B in nonsquamous cell lung cancer and its relationship with patient prognosis has not been determined. In our research, we demonstrated that SPRR1B is highly expressed in adenocarcinoma and is correlated with worse prognosis.

The SPRR family of proteins are encoded by polygene families gathered in the region of the epidermal differentiation complex. At present, the SPRR family consists of small proteins rich in proline: 2 SPRR1 (SPRR1A, SPRR1B), 7 SPRR2 (SPRR2A‐SPRR2F), and SPRR3 and SPRR4.^29^ Different proteins showed diverse organ‐ and tissue‐specific expressions. SPRR1 and 2 can be detected in the skin, conjunctiva, and oral cavity; SPRR3 can be detected in the oral cavity and esophageal epithelium; and SPRR4 can be detected in the intestine.^5^ In a study on the SPRR family and lung cancer, Xiong et al.^30^ showed that SPRR1A, SPRR1B, SPRR2D, SPRR2E, and SPRR3 are all highly expressed in the H1299 cell line. Oncomine also showed, in our analysis, that SPRR1B and SPRR2D are overexpressed in lung cancer. Moreover, in our volcano plot of high‐ and low expression SPRR1B groups, SPRR2D, SPRR3, SPRR1A, SPRR2A, SPRR2C, SPRR2E, and SPRR2F are among the most upregulated genes (Figure 8(a)). It is recommended that the relationship between the SPRR family genes and lung cancer should be studied in depth.

There have been several reports that SPRR1B plays an important role in carcinogenesis and proliferative behavior in cells.^26^ which was also confirmed in the present study. According to the results of the EdU proliferation assay and the colony formation assay, decreased expression of SPRR1B inhibited lung adenocarcinoma proliferation, induced G2/M‐phase arrest, and promoted apoptosis, which differs from the reported enhanced entry of cells into the G0 phase discussed by Tesfaigzi et al.^31^ Simultaneously, SPRR1B knockdown inhibited lung cancer cell migration and invasion, which indicates the potential role of SPRR1B in tumor invasion and metastasis, although further study is also needed.

Previous studies have demonstrated that the SPRR1B promoter is occupied by JunD and Fra2, and overexpression of fra1 in malignant cells enhanced SPRR1B promoter activity in malignant bronchial epithelial cells.^26^ Reddy et al. and Vuong et al. reported that SPRR1B transcription is mainly mediated by activator protein–1 (AP‐1), c‐Jun N‐terminal kinase‐1 (JNK1),^32^ and the activation of the MEK5‐ERK5 MAP kinase pathway.^33^ Michifuri et al. reported that SPRR1B can regulate the MAP kinase signal transduction pathway in oral squamous cell carcinoma stem‐cell‐like cells, thereby affecting cell growth through the suppression of Ras association domain family member 4 (RASSF4).^22^ Our GSEA data analysis also revealed that the MAP kinase signaling pathway was highly enriched in lung adenocarcinoma, which was validated through immunoblotting. Further investigation will determine whether RASSF4 is the direct target of SPRR1B in lung adenocarcinoma, and further studies are needed to discover the molecular mechanisms involved.

In conclusion, SPRR1B is highly expressed in lung adenocarcinoma, may predict poor prognosis, and is involved in diverse cell biological functions. Additional experiments are needed to explore the molecular mechanisms in more detail. In addition, further studies should be investigated. The associations of SPRR1B with clinicopathological features including smoking, the TNM stage, and nodal metastasis, need to be validated by multicenter studies.

## CONFLICT OF INTEREST

No authors report any conflict of interest.

## References

[1] Bunn PA Jr . Karnofsky award 2016: a lung cancer journey, 1973 to 2016. J Clin Oncol. 2017;35:243–52. 28056194 10.1200/JCO.2016.70.4064

[2] Hirsch FR , Scagliotti GV , Mulshine JL , Kwon R , Curran WJ Jr , Wu YL , et al. Lung cancer: current therapies and new targeted treatments. Lancet. 2017;389:299–311. 27574741 10.1016/S0140-6736(16)30958-8

[3] Li CY , Cai JH , Tsai J , Wang C . Identification of hub genes associated with development of head and neck squamous cell carcinoma by integrated bioinformatics analysis. Front Oncol. 2020;10:681. 32528874 10.3389/fonc.2020.00681 PMC7258718

[4] Gibbs S , Fijneman R , Wiegant J , van Kessel AG , van De Putte P , Backendorf C . Molecular characterization and evolution of the SPRR family of keratinocyte differentiation markers encoding small proline‐rich proteins. Genomics. 1993;16:630–7. 8325635 10.1006/geno.1993.1240

[5] Carregaro F , Stefanini AC , Henrique T , Tajara EH . Study of small proline‐rich proteins (SPRRs) in health and disease: a review of the literature. Arch Dermatol Res. 2013;305:857–66. 24085571 10.1007/s00403-013-1415-9

[6] Langfelder P , Horvath S . WGCNA: an R package for weighted correlation network analysis. BMC Bioinformatics. 2008;9:559. 19114008 10.1186/1471-2105-9-559 PMC2631488

[7] Wan Q , Tang J , Han Y , Wang D . Co‐expression modules construction by WGCNA and identify potential prognostic markers of uveal melanoma. Exp Eye Res. 2018;166:13–20. 29031853 10.1016/j.exer.2017.10.007

[8] Wang CCN , Li CY , Cai JH , Sheu PC , Tsai JJP , Wu MY , et al. Identification of prognostic candidate genes in breast cancer by integrated Bioinformatic analysis. J Clin Med. 2019;8:1160. 10.3390/jcm8081160 PMC6723760 31382519

[9] Szklarczyk D , Gable AL , Lyon D , Junge A , Wyder S , Huerta‐Cepas J , et al. STRING v11: protein‐protein association networks with increased coverage, supporting functional discovery in genome‐wide experimental datasets. Nucleic Acids Res. 2019;47:D607–13. 30476243 10.1093/nar/gky1131 PMC6323986

[10] Chin CH , Chen SH , Wu HH , Ho CW , Ko MT , Lin CY . cytoHubba: identifying hub objects and sub‐networks from complex interactome. BMC Syst Biol. 2014;8(Suppl 4)):S11. 25521941 10.1186/1752-0509-8-S4-S11 PMC4290687

[11] Shannon P , Markiel A , Ozier O , Baliga NS , Wang JT , Ramage D , et al. Cytoscape: a software environment for integrated models of biomolecular interaction networks. Genome Res. 2003;13:2498–504. 14597658 10.1101/gr.1239303 PMC403769

[12] Zhang C , Zhang Z , Zhang G , Zhang Z , Luo Y , Wang F , et al. Clinical significance and inflammatory landscapes of a novel recurrence‐associated immune signature in early‐stage lung adenocarcinoma. Cancer Lett. 2020;479:31–41. 32201203 10.1016/j.canlet.2020.03.016

[13] Rhodes DR , Yu J , Shanker K , Deshpande N , Varambally R , Ghosh D , et al. ONCOMINE: a cancer microarray database and integrated data‐mining platform. Neoplasia. 2004;6:1–6. 15068665 10.1016/s1476-5586(04)80047-2 PMC1635162

[14] Subramanian A , Tamayo P , Mootha VK , Mukherjee S , Ebert BL , Gillette MA , et al. Gene set enrichment analysis: a knowledge‐based approach for interpreting genome‐wide expression profiles. Proc Natl Acad Sci U S A. 2005;102:15545–50. 16199517 10.1073/pnas.0506580102 PMC1239896

[15] Li Y , Zhang H , Li Y , Zhao C , Fan Y , Liu J , et al. MiR‐182 inhibits the epithelial to mesenchymal transition and metastasis of lung cancer cells by targeting the met gene. Mol Carcinog. 2018;57:125–36. 28940757 10.1002/mc.22741

[16] Guo YJ , Pan WW , Liu SB , Shen ZF , Xu Y , Hu LL . ERK/MAPK signalling pathway and tumorigenesis. Exp Ther Med. 2020;19:1997–2007. 32104259 10.3892/etm.2020.8454 PMC7027163

[17] Khozin S , Miksad RA , Adami J , Boyd M , Brown NR , Gossai A , et al. Real‐world progression, treatment, and survival outcomes during rapid adoption of immunotherapy for advanced non‐small cell lung cancer. Cancer. 2019;125:4019–32. 31381142 10.1002/cncr.32383 PMC6899461

[18] Cortinovis D , Abbate M , Bidoli P , Capici S , Canova S . Targeted therapies and immunotherapy in non‐small‐cell lung cancer. Ecancermedicalscience. 2016;10:648. 27433281 10.3332/ecancer.2016.648 PMC4929979

[19] Díaz‐Serrano A , Gella P , Jiménez E , Zugazagoitia J , Paz‐Ares Rodríguez L . Targeting EGFR in lung cancer: current standards and developments. Drugs. 2018;78:893–911. 29915896 10.1007/s40265-018-0916-4

[20] Xiang M , Zhang W , Wen H , Mo L , Zhao Y , Zhan Y . Comparative transcriptome analysis of human conjunctiva between normal and conjunctivochalasis persons by RNA sequencing. Exp Eye Res. 2019;184:38–47. 30999002 10.1016/j.exer.2019.04.005

[21] Trzeciak M , Sakowicz‐Burkiewicz M , Wesserling M , Dobaczewska D , Gleń J , Nowicki R , et al. Expression of cornified envelope proteins in skin and its relationship with atopic dermatitis phenotype. Acta Derm Venereol. 2017;97:36–41. 27304082 10.2340/00015555-2482

[22] Michifuri Y , Hirohashi Y , Torigoe T , Miyazaki A , Fujino J , Tamura Y , et al. Small proline‐rich protein‐1B is overexpressed in human oral squamous cell cancer stem‐like cells and is related to their growth through activation of MAP kinase signal. Biochem Biophys Res Commun. 2013;439:96–102. 23954638 10.1016/j.bbrc.2013.08.021

[23] Liu Y , Zhu D , Xing H , Hou Y , Sun Y . A 6‐gene risk score system constructed for predicting the clinical prognosis of pancreatic adenocarcinoma patients. Oncol Rep. 2019;41:1521–30. 30747226 10.3892/or.2019.6979 PMC6365694

[24] Sheng Z , Han W , Huang B , Shen G . Screening and identification of potential prognostic biomarkers in metastatic skin cutaneous melanoma by bioinformatics analysis. J Cell Mol Med. 2020;24:11613–8. 32869947 10.1111/jcmm.15822 PMC7576265

[25] Oyesanya RA , Bhatia S , Menezes ME , Dumur CI , Singh KP , Bae S , et al. MDA‐9/Syntenin regulates differentiation and angiogenesis programs in head and neck squamous cell carcinoma. Onco Targets Ther. 2014;1:725–37. 10.18632/oncoscience.99 PMC4278274 25593999

[26] Patterson T , Vuong H , Liaw YS , Wu R , Kalvakolanu DV , Reddy SP . Mechanism of repression of squamous differentiation marker, SPRR1B, in malignant bronchial epithelial cells: role of critical TRE‐sites and its transacting factors. Oncogene. 2001;20:634–44. 11313996 10.1038/sj.onc.1204134

[27] Tesfaigzi J , Carlson DM . Expression, regulation, and function of the SPR family of proteins. A review. Cell Biochem Biophys. 1999;30:243–65. 10356644 10.1007/BF02738069

[28] Tesfaigzi J , Wright PS , Oreffo V , An G , Wu R , Carlson DM . A small proline‐rich protein regulated by vitamin a in tracheal epithelial cells is induced in lung tumors. Am J Respir Cell Mol Biol. 1993;9:434–40. 8398182 10.1165/ajrcmb/9.4.434

[29] Reddy SP , Vuong H , Adiseshaiah P . Interplay between proximal and distal promoter elements is required for squamous differentiation marker induction in the bronchial epithelium: role for ESE‐1, Sp1, and AP‐1 proteins. J Biol Chem. 2003;278:21378–87. 12682075 10.1074/jbc.M212258200

[30] Xiong Y , Li M , Zhang P , Zhang L , Yang Y . Study on Genetype in lung squamous carcinoma by high‐throughput of transcriptome sequence. Zhongguo Fei Ai Za Zhi. 2017;20:727–31. 29167000 10.3779/j.issn.1009-3419.2017.11.01 PMC5973275

[31] Tesfaigzi Y , Wright PS , Belinsky SA . SPRR1B overexpression enhances entry of cells into the G0 phase of the cell cycle. Am J Physiol Lung Cell Mol Physiol. 2003;285:L889–98. 12832281 10.1152/ajplung.00065.2003

[32] Vuong H , Patterson T , Adiseshaiah P , Shapiro P , Kalvakolanu DV , Reddy SP . JNK1 and AP‐1 regulate PMA‐inducible squamous differentiation marker expression in Clara‐like H441 cells. Am J Physiol Lung Cell Mol Physiol. 2002;282:L215–25. 11792626 10.1152/ajplung.00125.2001

[33] Reddy SP , Adiseshaiah P , Shapiro P , Vuong H . BMK1 (ERK5) regulates squamous differentiation marker SPRR1B transcription in Clara‐like H441 cells. Am J Respir Cell Mol Biol. 2002;27:64–70. 12091247 10.1165/ajrcmb.27.1.20020003oc

